# A Critical Review of the CRISPR-Cas Technology in the Detection of SARS-CoV-2 Variants

**DOI:** 10.1155/cjid/9107724

**Published:** 2025-07-09

**Authors:** Jie Zhang, Juezhuo Li, Jiawei Zhou, Jiaye Zhong, Yue Xu, Xiaolei Mao, Minghui Xu, Shuyin Luo, Yi Yang, Ruiyao Hu, Dong-Ang Liu, Shiyu Chen, Yuting Qiu, Keyi Chen, Jinghua Yuan, Xinling Zhang, Xiaoping Li

**Affiliations:** ^1^Key Laboratory of Artificial Organs and Computational Medicine in Zhejiang Province, Shulan International Medical College, Zhejiang Shuren University, Hangzhou, China; ^2^Wycombe Abbey School Hangzhou, Hangzhou, China

**Keywords:** COVID-19, CRISPR-Cas system, detection, mutant strain, SARS-CoV-2

## Abstract

Coronavirus disease 2019 (COVID-19) caused by severe acute respiratory syndrome coronavirus 2 (SARS-CoV-2) is still going on, and as the epidemic situation continues, the genome of SARS-CoV-2 is also mutating and evolving, resulting in more and more SARS-CoV-2 mutant strains, which have brought serious pressure on the prevention and control of COVID-19. Given that the COVID-19 is still spreading, it is extremely important to rapidly identify SARS-CoV-2 variants by nucleic acid assays. Thus, developing highly sensitive and specific assays that are suitable for field testing, high-throughput, and automation, as well as other diagnostic applications for SARS-CoV-2 variants, is urgently needed. This paper reviews the research progress of novel CRISPR-based diagnostic methods for SARS-CoV-2 variants.

## 1. Introduction

### 1.1. Status of the Coronavirus Disease 2019 (COVID-19) Epidemic

COVID-19 is a global pandemic caused by severe acute respiratory syndrome coronavirus 2 (SARS-CoV-2) [1], which has been included in the scope of category B infectious diseases in China.

SARS-CoV-2 has some pathogenicity. Most patients present with mild symptoms after infection with SARS-CoV-2, including cough, fever, body weakness, and muscle aches and pains [2, 3]. Additionally, there may be some patients with asymptomatic infection [4]. However, approximately 20% of COVID-19 patients develop severe symptoms, including respiratory distress and hypoxemia [5].

From the start of the pandemic until November 10, 2024, over 776.8 million confirmed COVID-19 cases and over 7 million confirmed deaths were notified to the World Health Organization (WHO) across 234 countries. The majority of COVID-19-associated deaths occurred in 2020, 2021, and 2022, with increased immunity leading to a significant decrease in deaths. For the latest 4 weeks reporting period, from 14 October to 10 November 2024, 77 countries reported COVID-19 cases and 27 deaths globally. The number of reported cases decreased by 39%, with over 200,000 new cases and 36% of new deaths, compared to the previous 28 days. The WHO is monitoring several SARS-CoV-2 variants, including one variant of interest (VOI) JN.1 [6].

In China, 90.58% of the Chinese population have received COVID-19 vaccines and the situation of COVID-19 is generally stable [7]. However, it still poses considerable difficulties for doctors in detecting and judging COVID-19 due to its mutable and evolvable characteristics. Therefore, we need to research new methods to identify and detect SARS-CoV-2 variants.

### 1.2. Evolution Trend and Harm of SARS-CoV-2 Variants

The global COVID-19 pandemic is mainly attributed to continuous evolutions and genomic mutations of SARS-CoV-2. The viral mutations have caused differences in its genetic structure, as well as affecting its functional activity, such as enhancing its viral infectivity and viral stealth ability, decreasing its serum neutralizing activity in humans, and enhancing its immune escape ability. As a result, existing vaccines are less protective and the current global epidemic is difficult to control and treat [8].

Between September 2020 and September 2023, a variety of SARS-CoV-2 mutant strains have emerged. They have mutated the S gene in various combinations, leading to a notable rise in the infectivity and pathogenicity of SARS-CoV-2 and altering its immunogenicity to some degree [8, 9]. Some of the mutated strains were classified as “variants of concern” (VOCs) by the WHO at the beginning of 2021. They have the following characteristics: increased transmission and infectious ability of the viral strains and increased viral pathogenicity and infectivity. Several mutant strains have been identified, and according to the WHO definition, there are five mutant strains belonging to VOC (Table 1). They include the mutant B.1.1.7 (alias 501Y.V1, also known as Alpha), the mutant B.1.351 (alias 501Y.V2, also known as Beta), the mutant P.1 (alias 501Y.V3, also known as Gamma), the mutant B.1.617.2 (also known as Delta), and the mutant B.1.1.529 (also known as Omicron) [10]. The appearance of each VOC triggers widespread SARS-CoV-2 outbreaks, significantly challenging and complicating efforts to prevent and manage these epidemics. Studies have shown that the HV69-70del mutation site is the key mutation site in Alpha and Omicron mutant strains [11]. A global pandemic of COVID-19 remains ongoing, and the risk of new pathogenic variants remains constant; it is essential to develop methods that can detect new variants early and accurately [12]. There are many methods widely used for SARS-CoV-2 variant diagnosis; we have compared the advantages and disadvantages of existing detection methods and CRISPR-Cas–based methods [13] (Table 2).

## 2. Existing Detection Methods for SARS-CoV-2 Variants

With the persistent epidemic of COVID-19 and the continuous mutation of SARS-CoV-2, in the prevention and control of COVID-19, virus transmission control, detection of infectious agents, and isolation of infected people, nucleic acid detection plays a particularly important role. Examples include genome sequencing technology, real-time fluorescence quantitative RT-PCR technology, and microdroplet digital PCR technology. These technologies have made important contributions to China's epidemic prevention and control. Each nucleic acid detection technique has its strengths and weaknesses and can be used together to monitor SARS-CoV-2 and its variants with greater precision. Currently, the following nucleic acid tests are available for the SARS-CoV-2 variants.

### 2.1. Genome Sequencing Technology

**Figure 1:**
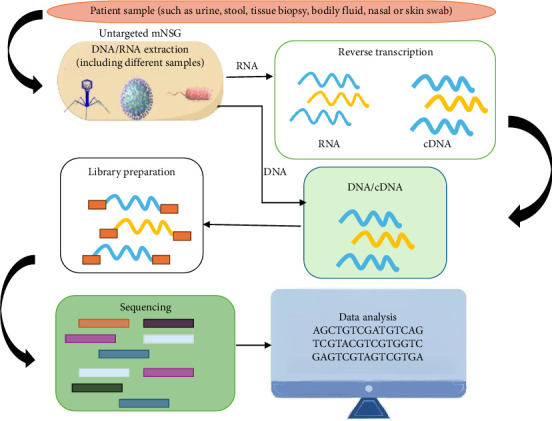
Working mechanism for mNGS. Footnote: ①Sample collection: Various patient samples (e.g., urine, stool, tissue biopsy, bodily fluids, and nasal/swab) are collected. ②Nucleic acid extraction: DNA/RNA extraction: Both DNA and RNA are isolated from the sample, with methods tailored to different sample types. Reverse transcription (for RNA): RNA is converted to complementary DNA (cDNA) to enable sequencing. ③Library preparation: DNA and cDNA are processed into sequencing libraries, which involve fragmentation, adapter ligation, and amplification to prepare the genetic material for sequencing. ④ Sequencing: High-throughput NGS platforms sequence the prepared libraries, generating vast amounts of short reads (e.g., AGCTGTCG…). ⑤Data analysis: Bioinformatics tools analyze the raw sequences to identify pathogens by comparing reads to genomic databases. Detect novel or unknown microbes through alignment and assembly. Quantify microbial abundance and assess antimicrobial resistance or virulence genes.

Gene sequencing often employs mNGS, which stands for metagenomics next generation sequencing [14, 15]. In recent years, with the development of emerging molecular biology detection technology, mNGS detection of clinical pathogenic microorganisms has gradually increased in research reports [19]. In a typical mNGS workflow, a clinical sample is first acquired, followed by RNA or DNA extraction. High-throughput sequencing is then carried out, sequencing nucleic acid fragments from the library using a chosen platform (Figure 1). For example, early in the COVID-19 outbreak, Zhu et al. used mNGS to parse the first SARS-CoV-2 genome sequence [20]. At the same time, mNGS provides important public health guidance for identifying origin of viral outbreaks and assisting in monitoring the evolution of the virus during the epidemic prevention and control period. Zhou et al. used gene sequencing to construct an evolutionary tree to reveal SARS-CoV-2-related properties, analyzed the natural host traceability of the virus, the path of virus transmission to humans, the pathogenic pathological mechanism of the virus, etc., and pointed out that more in-depth studies are needed to investigate the impact of SARS-CoV-2 on human health after mutation in animals [21]. mNGS technology can identify new mutant strains and track VOC in real time; however, it is complex and time consuming, and the cost investment is high [22]. At the same time, some targeted NGS (tNGS) technologies have also been used to detect SARS-CoV-2. The ARTIC Network has developed a widely used protocol for SARS-CoV-2 sequencing using Oxford Nanopore Technologies (ONT) and Illumina platforms. The protocol is optimized for generating high-quality, amplicon-based whole-genome sequences from clinical samples [23]. As an example, Integrated DNA Technologies (IDT) has been employed to identify SARS-CoV-2, being both simple and highly efficient [24].

### 2.2. Real-Time Fluorescence Quantitative Polymerase Chain Reaction (RT-fqPCR) Technology

**Figure 2:**
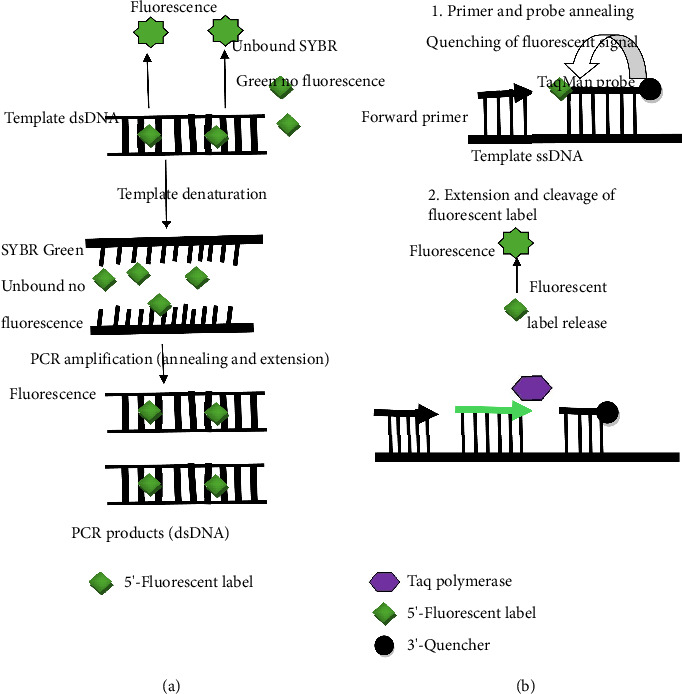
Working mechanism for RT-fqPCR. (a) SYBR Green assays. (b) TaqMan (5′ nuclease) assays. Footnote: SYBR green assays: ① Denaturation: High temperature (∼95℃) causes the double-stranded DNA template (dsDNA) to unwind into ssDNA. The free SYBR Green dye does not emit fluorescence when it is not bound to DNA. ②Annealing and extension: As the temperature drops, the primers specifically bind to the template DNA (Annealing). DNA polymerase synthesizes complementary strands starting from primers to form new dsDNA (extension). The SYBR Green binds to the newly synthesized dsDNA and emits fluorescence signals. ③Fluorescence detection: During each PCR cycle, the fluorescence signal increases with the accumulation of dsDNA products, thereby enabling real-time quantitative measurement. TaqMan assays: ①Denaturation and annealing: Double-stranded DNA shifts from a double-stranded to a single-stranded state. The forward primer and TaqMan probe simultaneously attach to the template ssDNA. When the probe is intact, the fluorescent group has no signal because the quenching group is close to it. ②Extension and cleavage: Taq DNA polymerase synthesizes new strands along the template. When encountering the probe, it exhibits 5'⟶3' exonuclease activity and cleaves the probe. The fluorescent group and the quenching group are separated, releasing the fluorescence signal. ③Fluorescence detection: The cumulative fluorescence signal of each round of cutting event is proportional to the amount of PCR products.

RT-fqPCR is a method for detecting RNA. Especially in the detection of viruses and gene expression analysis, it is capable of quantitatively assessing the level of expression of specific genes and can be used as a transcriptome sequencing analysis of a more accurate technical complement [16]. By combining reverse transcription (RT) and real-time fluorescent PCR, this technique can reverse RNA and amplify DNA simultaneously and monitor PCR products in real time (Figure 2). RT-fqPCR is relatively simple to perform and offers a wide dynamic range of assays with high sensitivity and specificity, as well as the possibility of quantitative analysis to determine the initial values of the RNA. The advantage of RT-fqPCR over mNGS is speed and economics. However, RT-fqPCR still requires specialized fluorescence equipment and professional detection personnel [25].

SARS-CoV-2 detection can be divided into two levels: virus detection and variant detection. Virus detection is usually based on RT-fqPCR, targeting conserved regions of the genome (such as N gene or ORF1ab), and the method is stable and reliable, which is suitable for clinical diagnosis and large-scale screening. Variant detection is more challenging, which requires sequencing or specific mutation detection to identify key mutations (such as spike protein mutations). However, due to the continuous evolution of the virus, the detection methods may need to be continuously optimized. Therefore, conventional virus detection is less affected by variation, while variant surveillance requires dynamic adjustment of strategies to adapt to emerging variant strains [26].

### 2.3. Droplet Digital PCR (ddPCR)

**Figure 3:**
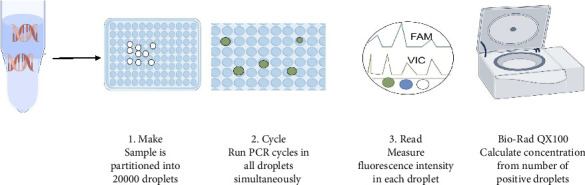
Working mechanism for ddPCR. ①Make: After mixing the sample to be tested with the PCR reaction mixture, the sample is divided into approximately 20,000 independent droplets by a droplet generator. Each droplet is equivalent to an independent PCR reaction unit, which may or may not contain the target nucleic acid molecules. ②Cycle: All microdroplets undergo PCR cycles (denaturation, annealing, and extension) simultaneously. Since the microdroplets are independent of each other, the amplification process is not interfered with. Microdroplets containing the target nucleic acid will undergo specific amplification, while those without the target will have no amplification signal. ③Read: Measure the fluorescence intensity of each microdroplet individually using a fluorescence detection system. Microdroplets containing amplification products (positive) will show fluorescence signals, while those without will be negative. By counting the number of positive microdroplets and applying Poisson distribution correction, the initial concentration of the target nucleic acid can be accurately calculated. ④Data analysis and output: The instrument (such as Bio-Rad QX100) can automatically analyze data and output the absolute concentration of the target molecule without relying on standard curves. It has high sensitivity and accuracy.

ddPCR is an absolute quantitative method but has very cumbersome assay steps [17]. Introduce nucleic acid into the reaction system of highly dispersed droplets for each droplet in the PCR reaction, and then use fluorescence measurement to assess the increase in positive and negative signals (Figure 3). In comparison to ddPCR, RT-fqPCR is more suitable for detecting SARS-CoV-2 variants [27]. Although it has a much lower limit of detection (LoD), it relies on expensive equipment and consumables, leading to very highexpenses and making it unsuitable for on-site Point-of-Care Testing (POCT).

In addition, there are several other RT-PCR assays. Examples include TaqMan, Molecular Beacons, and so on. TaqMan probe method is a highly specific quantitative PCR technology. The core of the method is to use the 3′ to 5′ exonuclease activity of Taq enzyme to cut off the probe and generate fluorescence signal. Since the probe binds specifically to the template, the intensity of the fluorescence signal represents the amount of template [28]. The Molecular Beacons were established by Tyagi and Kramer. The probe was single-stranded DNA (ssDNA) and labeled with reporter and quenched fluorophores at the 5′ and 3′ ends of the probe, respectively. It is mainly used for mutation detection, pathogen quantification, virus replication, and fetal sex detection [28, 29].

### 2.4. Lateral Flow Immunoassay (LFIA)

LFIA has the advantage of simple operation and low cost, so researchers have combined CRISPR assays with LFIA to establish a variety of on-site assays that do not require fluorescent reads [30–32]. For example, Kumar et al. used the high specificity of FnCas9 to identify mismatches in the target and developed a CRISPR detection method based on LFIA (FnCas9 editor linked uniform detection assay, FELUDA) [33]. On the other hand, Ali et al. established a biotin-coupled specific CRISPR-based assay for nucleic acid detection (Bio-SCAN) using LFIA in combination with biotin-tagged dCas9 [31]. This method can effectively distinguish SARS-CoV-2 Alpha, Beta, and Delta within 1 h by reverse transcription recombinase polymerase amplification (RT-RPA) taking 15 min and CRISPR detection taking 30 min. He et al. combined RT-PCR amplification with CRISPR-Cas12a detection to establish a field-applicable assay, which was named RT-CORDS (Cas12a-based RT-PCR combined with CRISPR on-site rapid detection system) [32]. It can detect 10–16 M (about 16,000 copies/mL) of SARS-CoV-2 nucleic acid using LFIA.

### 2.5. Other Nucleic Acid Assays Based on Isothermal Amplification

Nucleic acid detection based on isothermal amplification technology is a new type of nucleic acid detection method in recent years, which can realize rapid amplification of trace nucleic acids in a relatively short period (30–40 min) without the need for sophisticated temperature control equipment [34]. In order to detect SARS-CoV-2 variants rapidly, researchers use loop-mediated isothermal amplification (LAMP), recombinase-aided amplification (RAA), and other techniques [35, 36]. RAA and other techniques are suitable for rapid amplification in the field, but they are prone to false positives due to nonspecific amplification and have great limitations.

## 3. CRISPR-Based Nucleic Acid Detection of SARS-CoV-2 Variants

### 3.1. Overview of the CRISPR-Cas System

In the CRISPR-Cas system, CRISPR and Cas proteins work in tandem to form the effector complex, which cleaves nucleic acids and recognizes them [37]. CRISPR-Cas system is essentially bacterium's weapon to defend against foreign DNA (e.g., phage) by cleavage of such DNA.

Different Cas proteins have different nucleic acid endonuclease activities. The current studies focus mainly on Cas9, Cas12, Cas13, and Cas14 (Table 3). Cas9 binds to single-guide RNA (sgRNA) and then specifically identifies and cuts the target, and target detection is achieved through the analysis of cut products [38]. On the other hand, Cas12, Cas13, and Cas14 form an effector complex after recognizing and binding to CRISPR RNA (crRNA) and target, which can nonspecifically cleave any ssDNA or single-stranded RNA (ssRNA), and this process is called trans-cleavage. These proteins trans-cleave many labeled ssDNA or ssRNA reporter probes, which in turn outputs a detection signal. Pathogenic microbial detection technologies based on the CRISPR-Cas system can be divided into two categories [39]: In the first category, nucleic acid detection methods are developed using microbial gene targets and CRISPR-Cas technology; in the second category, aptamers or antibodies are used to detect nonnucleic acid analyses and CRISPR-Cas is used to recognize nonnucleic acid analyses.

**Figure 4:**
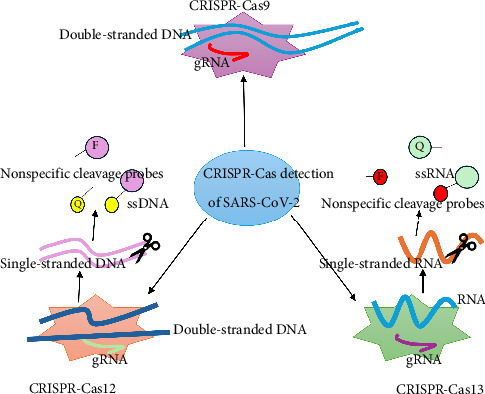
Various types of CRISPR-Cas systems applied for detecting SARS-CoV-2 variants. Footnote: F: fluorescein amidite (FAM) Q: black hole quenching dye-1 (BHQ-1) CRISPR-Cas9 System: Targets double-stranded DNA (dsDNA) using a guide RNA (gRNA). Performs nonspecific cleavage of the target, which can be detected using probes. Output includes ssDNA or other byproducts for analysis. CRISPR-Cas12 System: Also targets double-stranded DNA (dsDNA) or ssDNA. Activates collateral cleavage of nearby nontarget ssDNA (probes), enabling highly sensitive detection. Output signals are amplified through probe degradation. CRISPR-Cas13 System: Unique in targeting ssRNA, making it ideal for RNA viruses like SARS-CoV-2. Exhibits collateral cleavage of surrounding RNA probes, similar to Cas12 but for RNA. Output includes cleaved RNA fragments for signal readout.

At present, many CRISPR-Cas systems have been developed to detect SARS-CoV-2. With the continuation of the epidemic, SARS-CoV-2 is also mutating and evolving. As a result of mutations in multiple loci in the SARS-CoV-2 genome, the virus is more capable of transmitting and evading immunity, making prevention and control extremely difficult. To achieve highly specific detection of the SARS-CoV-2 variants, researchers have optimized the CRISPR system in various ways to improve the specificity of the CRISPR-based novel coronavirus nucleic acid detection technology. They developed the SARS-CoV-2 variant nucleic acid detection technology suitable for different application scenarios, such as on site, high-throughput, and automated, which provides rapid monitoring of the SARS-CoV-2 variants [31–33, 38–47] (Table 4). The main ones applied to the detection of SARS-CoV-2 variants include CRISPR-Cas9, CRISPR-Cas12, and CRISPR-Cas13 [46, 47] (Figure 4). Among them, CRISPR-Cas9 recognizes and cleaves target DNA/RNA sequences by the Cas9 protein under the guidance of guide-RNA (gRNA) [48]; CRISPR-Cas12 recognizes target nucleic acid sequences under the guidance of gRNA and activates the targeted and untargeted cleavage activity of Cas12 [46, 49]; CRISPR-Cas13 recognizes and cuts targeted ssRNA under the guidance of gRNA while activating the nontargeted cutting ssRNA function of Cas13 [50]; CRISPR-Cas14 recognizes and cuts targeted ssDNA under the guidance of gRNA.

### 3.2. CRISPR-Based Rapid On-Site Detection of SARS-CoV-2 Mutant Loci

To improve the detection of mutants in the grassroots and underdeveloped areas, several CRISPR-based visual fluorescent SARS-CoV-2 mutation site detection methods have been developed [44, 51]. Marques et al. developed an on-site detection method for the SARS-CoV-2 E484K mutation using RT-PCR and CRISPR-Cas12a [52]. They designed a specific sequence near the mutation site recognized by Cas12a, which produces a visual fluorescence signal. It was even shown that RT-RPA, combined with the CRISPR-Cas12aenzymatic reaction method, could detect novel coronavirus variants—includingkey mutants such as Alpha, Beta, Gamma, Delta, Lambda, Mu, Kappa, Omicron, andtheir sub-variants BA.1, BA.2, BA.3, BA.4, and BA.5. They can be detected instantly with high throughput in less than 60 min, and these SARS-CoV-2 positive samples have viral titers between 10^4^ and 10^8^ copies/reaction [53–56]. However, the specificity of CRISPR-based assays must be carefully validated to avoid cross-reactivity with nontarget variants. For example, the E484K-targeting Cas12a system may also detect E484Q or E484P mutations if the gRNA design is not stringent [52]. Recent studies suggest that introducing synthetic mismatches in crRNAs (e.g., dual synthetic mismatch detection based on CRISPR-Cas12a (dsmCRISPR)) can improve single-nucleotide discrimination, reducing false positives [57].

For in situ detection of SARS-CoV-2 D614 G mutation sites, Ning et al. used crRNAs designed to target the PAM motif or the seed region of CRISPR-Cas12a [55]. In addition, they could detect 500 copies/mL of SARS-CoV-2 nucleic acid containing a specific variant in a system with a total reaction volume of 20 μL [58]. Fasching et al. used the CRISPR-Cas12 system to identify point mutations in 261 SARS-CoV-2 positive samples and used it as a comparison with WGS [59]. The results showed that the method had 98.9% lineage classification agreement with WGS, indicating that the CRISPR-Cas12 system was able to accurately identify SARS-CoV-2 variants. Notably, the 1.1% discrepancy between CRISPR and WGS in Fasching's study may stem from off-target effects or low viral load samples [59]. Future assays could incorporate computational tools (e.g., CRISPR off) to predict and minimize off-target gRNA binding.

Liang et al. established a field-based detection method for SARS-CoV-2 Omicron variants using double crRNA with three mismatches, which also used visual fluorescence [60]. This method also used visual fluorescence for the reading of results, enhancing the assay'sspecificity and sensitivity, and detecting 2 copies/reaction of plasmid DNAfrom SARS-CoV-2 Omicron variants. The double-crRNA strategy effectively reduces off-target risks but may increase assay complexity [60]. A balance between multiplexing and simplicity is critical for field applications. Furthermore, Huang et al. established dsmCRISPR for the SARS-CoV-2 D614 G variant site by designing a CRISPR-Cas12a–based in situ detection method, which can achieve rapid reading results by simple UV irradiation [57]. With excellent results, Luo et al. used RT-RPA along with CRISPR-Cas12a to validate the detection of variants Omicron BA.2, BA.4, and BA.5 [61].

Doudna's research team developed a new nucleic acid detection technique called fast integrated nuclease detection in tandem (FIND-IT) [18]. Her research team published Version 1.0 of this technology by using Cas13 [62]. Following the cutting of RNA, nonspecific ssRNA with fluorescent markers is turned into a fluorescent signal to evaluate the result, reaching a sensitivity of 100 copies/μL in 30 min.

The all-in-one dual CRISPR-Cas12a (AIOD-CRISPR) is an innovative diagnostic tool designed for the rapid and highly sensitive detection of nucleic acids, such as viral RNA or DNA, including from pathogens like SARS-CoV-2 variants [63].

One-hour low-cost multipurpose highly efficient system (HOLMES) against SARS-CoV-2 variants is designed to quickly and affordably detect the virus. The method employs CRISPR-based diagnostics or LAMP technology to detect SARS-CoV-2 variants in under an hour, offering high sensitivity and specificity at an affordable price. Additionally, this system would be accessible in resource-limited settings, offering a scalable solution for both diagnostics and treatment. HOLMES aims to reduce transmission rates and improve patient outcomes through its rapid, multipurpose approach [64].

### 3.3. CRISPR-Based High-Throughput Multitargeting

To realize the simultaneous detection of multiple mutation sites of SARS-CoV-2 variants and then realize the large-scale rapid screening of SARS-CoV-2 variants, researchers have established CRISPR-based high-throughput multitargeted SARS-CoV-2 variant detection methods in various ways. For example, Shinoda et al. developed an automated amplification-free digital RNA detection platform (open-SATORI) using CRISPR-Cas13a detection technology and a microcavity device [65]. The open-SATORI platform can automate the process from sample mixing to RNA quantification in clinical samples in 9 min. SARS-CoV-2 genomic RNA of 6.5 aM (approximately 3.9 copies/μL) was detected in a reaction volume of 105 μL, and it can accurately differentiate between SARS-CoV-2 variants, including Alpha, Delta, and Omicron mutants. Therefore, open-SATORI can be used as a rapid diagnostic platform for the identification of SARS-CoV-2 variants, and automated high-throughput detection of SARS-CoV-2 variants can be realized. While CRISPR enables high-throughput variant detection, its scalability depends on rapid assay adaptation to emerging variants. For instance, the open-SATORI platform can be reconfigured for new variants within days by replacing crRNAs, but clinical validation may require additional weeks [65]. Standardized crRNA design pipelines (e.g., bioinformatics tools like CHOPCHOP) could accelerate this process.

Ackerman et al. used the CRISPR-Cas13a assay to establish CARMEN, a combinatorial array reaction platform for multiplexed nucleic acid evaluation, to automate the detection of at least 10 human-associated viruses with currently known genome sequences, including SARS-CoV-2 variants. Recently, Myhrvold et al. established the microfluidic CARMEN (mCARMEN) assay, also known as the microfluidic combinatorial array reaction (MCAAR) [66]. CARMEN technology combines a microfluidic chip with an automated nucleic acid extraction device, which can be used to identify six viruses, including Delta and Omicron variants. Multiplexing assays like mCARMEN face trade-offs between throughput and specificity [66]. For example, simultaneous detection of Delta (L452R) and Omicron (S: R346T) requires orthogonal gRNAs to prevent crosstalk. Methodological validation must be experimentally verified using mixed variant samples. Using this method, multiple viral nucleic acids can be evaluated with results that are nearly identical to those of gene sequencing, allowing simultaneous monitoring of several viruses and their mutants.

The CRISPR-based SHERLOCK nucleic acid assay is considered a highly sensitive and rapid nucleic acid assay, which can be combined with LFIA to achieve rapid on-site detection of SARS-CoV-2 variants, but it still has a throughput limitation. Based on the SHERLOCK nucleic acid detection technology, Puig et al. developed a small-scale automated SHERLOCK nucleic acid detection device, called miSHERLOCK [67]. miSHERLOCK is able to use unprepared patient saliva directly as a sample because it has a small device that automatically extracts and purifies the viral RNA and uses RT-RPA and CRISPR-Cas13a reaction reagents for amplification and specific recognition, with the entire testing process completed in less than 1 h. miSHERLOCK is a compact, automated, and easy-to-use device for the detection of viral RNA. Multiplexed detection of mutation sites associated with SARS-CoV-2 and its mutants B.1.1.7, B.1.351, and P.1 is possible with the miSHERLOCK [54]. The device also enables the quantification of the result output, automated analysis, and remote result reporting with the assistance of a smartphone application.

Cao et al. recently developed an automated, portable, and high-throughput fluorescence analyzer (APHF-analyzer) based on RT-RPA amplification with CRISPR-Cas13a detection [68]. The APHF-analyzer uses a 3D printed microfluidic chip to detect SARS-CoV-2 S and Orf1ab genes. It can detect concentrations as low as 0.68 fM within 30 min with a reaction system of 20 μL. The APHF-analyzer was combined with LFIA to detect 380 clinical samples, and the results showed high sensitivity and specificity [69]. The biosensor achieved visual detection, enabled to conduct home testing, and reduced work pressure in hospitals by using LFIA.

## 4. Discussion

The sudden appearance of SARS-CoV-2 has greatly affected people's daily lives, and the SARS-CoV-2 variants have also brought some troubles to people's lives. Currently, several methods have been used for the detection of SARS-CoV-2 variants. The nucleic acid detection system based on CRISPR-Cas can effectively transform the targeted DNA molecular signals into detection tools or fluorescent signals, chemical signals, electrochemical signals, etc., which can be observed by naked eyes to realize the detection of target nucleic acid signal molecules [64]. Using CRISPR-Cas system techniques to detect SARS-CoV-2 variants can greatly minimize the necessity for pricey testing equipment and time, thus reducing testing costs. This enables these technologies to be implemented in less affluent regions.

Compared with traditional molecular diagnostic techniques, the CRISPR-Cas nucleic acid assay has many advantages. First, the CRISPR-Cas assay does not require professional personnel, and it is simple to operate and saves time. For example, SHERLOCK can get results in about 35 min. Second,CRISPR-Cas nucleic acid detection technologies—such as SHERLOCK, which achievessingle-nucleotide resolution—serve as powerful tools for bacterial genotyping,identifying mutation sites in drug-resistant microorganisms, and detectingtumor-associated DNA methylation. Third, the CRISPR-Cas is compatible with various signal sensing systems, such as electrochemical sensors, solid-phase nanopore sensors, and LFIA. It provides more possibilities and diversities for the practical application of CRISPR-Cas. Fourth, the CRISPR-Cas system offers numerous advantages over the standard RT-fqPCR approach, boosting current molecular detection capabilities [70, 71]. Furthermore, CRISPR-Cas is suitable for diagnosing COVID-19 due to its rapid testing ability, ease of use, and portability. Unlike traditional methods such as PCR, which require an expensive thermal cycler, CRISPR-Cas employs a simple molecular mechanism to detect viral RNA and can generate results in as little as 30–60 min. Moreover, CRISPR-Cas can be incorporated into small, affordable devices that do not need sophisticated equipment, making it perfect for decentralized testing in clinics, airports, and remote areas. Its high sensitivity and specificity are comparable to those of PCR, ensuring that it can reliably identify low viral loads, which is essential for early detection. CRISPR-Cas systems can be engineered to identify several targets at once, enabling the detection of various SARS-CoV-2 genes and other respiratory pathogens in a single test [71, 72] (Table 5).

Although the CRISPR-Cas system is of great value in pathogen diagnosis, it still faces certain challenges in subsequent applications. First, due to the inherent off-target effect of CRISPR-Cas system, tolerance of Cas proteins to mismatches leads to easy off-target binding, which weakens the specificity of the method. Secondly,because both Cas9 and Cas12 proteins rely on PAM sequences to recognize andbind to target sequences. RT-fqPCR offers high versatility in pathogen detection, whereas CRISPR-Cas technology is constrained by specific PAM sequences or loci, limiting its capacity to detect diverse target sequences and hindering its broader application. To remove the dependence of the CRISPR-Cas system on PAM sequences, Ding et al. developed a double crRNA assay without PAM sequences based on the CRISPR-Cas12a assay and achieved highly sensitive detection of the SARS-CoV-2 N gene [74]. Their results showed that the detection of double crRNA without the PAM sequence was more sensitive than the detection of single crRNA with the PAM sequence. Third, various Cas proteins have different limitations due to their characteristics. Among them, the Cas9 protein can only recognize and cut target sequences in a targeted manner, without the signal amplification effect of the Cas12 protein. Cas13 protein–based nucleic acid assays require additional in vitro transcription, increasing experimental complexity. To degrade the preamplified dsDNA products into recognizable ssDNA, Cas14 requires T7 nuclease exonuclease, which increased its complexity. Fourth,nucleic acid detection utilizing Cas12 and Cas13 proteins relies on thetrans-cleavage activity of Cas proteins, which nonspecifically cleave anysingle-stranded nucleic acids. CRISPR-Cas cannot detect multiple target sequences in a single system by designing different fluorescent probes, as in RT-fqPCR. Single-gene detection using Cas proteins is prone to false negatives and shows reduced accuracy when targeting pathogens with multiple mutation sites. Fifth, the widespread adoption of CRISPR-Cas diagnostics is also hindered by the necessity of a reliable cold chain, as the reagents are prone to rapid degradation. Furthermore, enzyme activity shows considerable variability from one batch to another. Strict cold-chain requirements and variability in production are the main challenges to the wider commercial adoption of CRISPR-Cas for diagnostics. Afterward, CRISPR-Cas nucleic acid detection involves a separate nucleic acid preamplification process and multiple reagent additions and transfers. It will increase the risk of cross-contamination between samples by opening the cap several times during the experiment. Furthermore, the results of different assays are presented in different ways, and each step in the assay process may lead to changes in the results.

## 5. Conclusions and Outlook

**Figure 5:**
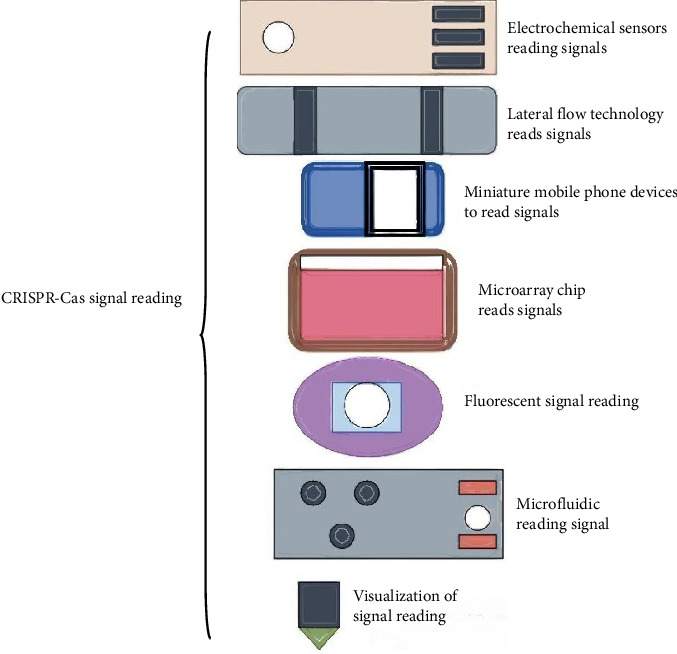
Signal readout for SARS-CoV-2 variant detection based on CRISPR-Cas system. Footnote: Electrochemical sensors: Measure changes in electrical properties (e.g., current or impedance) caused by the cleavage of reporter tags. Lateral flow technology: Uses labeled probes (e.g., gold nanoparticles) to produce visible lines on a strip, similar to rapid antigen tests. Miniature mobile phone devices: Capture and analyze signals (e.g., colorimetric or fluorescent changes) using smartphone cameras and apps for portable detection. Microarray chips: Detect fluorescent or colorimetric signals from multiple probes immobilized on a chip, enabling high-throughput variant screening. Fluorescent: Measures fluorescence emitted by cleaved probes using a fluorometer or imaging device. Microfluidic systems: Integrate sample processing and signal detection in miniaturized channels, enhancing sensitivity and automation. Visualization: Direct observation of color changes or precipitate formation by the naked eye for rapid, equipment-free results.

In recent years, the development of CRISPR-Cas tools has led to breakthroughs in the field of genome engineering. The discovery of Cas proteins has revolutionized the study of natural sciences and good progress has been made in basic research, therapeutics, and diagnostics. The ease of use, low equipment requirements, and low cost have made it possible for many laboratories to use these CRISPR-Cas–based systems to study gene function in a variety of organisms. During the COVID-19 epidemic, the CRISPR-Cas system was developed as a powerful nucleic acid diagnostic technology platform for the rapid detection of SARS-CoV-2 and its variants. It is considered a new generation of nucleic acid diagnostic technology with powerful programmability, high specificity, and high sensitivity. In addition, the CRISPR-Cas system meets the needs of various assays by combining it with other methods to present results (Figure 5), such as fluorescent signaling, visual readout based on UV light, ELISA, lateral flow assays, glycation test strips, morning-after test strips, electrochemical signaling, and microfluidic chip-based assays [62]. Multichannel high-throughput detection technology based on the CRISPR-Cas system realizes the simultaneous detection of SARS-CoV-2 and its multiple variants. The potential of CRISPR-Cas systems in the diagnosis of pathogens needs to be developed urgently [64].

However, we still lack sufficient understanding of many mechanisms of Cas proteins. Therefore, a deeper understanding of Cas proteins' molecular mechanisms, the identification of PAM-free Cas proteins, and the implementation of more precise targeting will significantly enhance Cas proteins' potential for genome engineering. Additionally, the advanced unexpressed Cas proteins that may be present in many bacteria or archaea will revolutionize multiple fields, including the diagnosis of novel viruses, therapeutics, agriculture, breeding, and more [75]. In the future, it would be helpful if these proteins could be fully understood for their improved genome editing abilities; the CRISPR-Cas detection system will need to be continuously optimized to reduce the number of steps and increase sensitivity. At the same time, it can be combined with more traditional assay techniques to improve specificity. By integrating multidisciplinary technologies such as materials science, automation engineering, computational science, and artificial intelligence with the CRISPR-Cas system, it is possible to improve the efficiency of the assay [76]. The CRISPR-Cas system will play an even more powerful role in pathogen diagnosis. If we can fully understand the improved genome editing ability of these Cas proteins and effectively improve the defects of CRISPR-Cas systems, it might launch a new CRISPR craze and result in groundbreaking and influential CRISPR technology emerging in the mainstream soon [73, 77].

## Figures and Tables

**Table 1:** Comparison of infectivity of major VOC of SARS-CoV-2.

VOC	Main feature	Domestic popularity time	Main mutation sites
Alpha	Highly infectious	First half of 2021	N501Y, HV69-70del, P601H
Beta	Strong immune escape	Not detected in China	N501Y, K417N, E484K
Gamma	High mortality rate	Not detected in China	N501Y, E484K, K417T
Delta	Highly infectious	Second half of 2021	L452R, P681R, T478K, D614G
Omicron	Highly infectious and invisible	2022 epidemic in parts of China	K417N, S477N, T478K, E484K, K417N, Q493R, N501Y, Y505H

**Table 2:** The advantages and disadvantages of existing detection methods and CRISPR-Cas–based methods.

Detection method	Advantages	Disadvantages	Application scenarios	References
Genome sequencing technology (mNGS)	Can detect any part of the genome, unbiased	Complicated and lengthy process, prone to contamination, expensive	Various sample types, including blood, cerebrospinal fluid, respiratory samples, and gastrointestinal fluid	[13–15]
Real-time fluorescence quantitative PCR technology	High sensitivity and accuracy, absolute and relative quantification, low risk of contamination	Risk of false-positive or negative detection, primers, and reaction efficiency can affect testing outcome	Standard PCR specialist laboratory	[13, 16]
Droplet digital PCR	High sensitivity and accuracy, absolute detection	Too specific, limiting virus use	Quantitation of gene copy number variation, gene expression, RNA/microRNA quantitation, and rare sequence detection	[13, 17]
CRISPR-based detection methods	Ultrasensitive, high specificity, rapid analysis	Multistep process is prone to contamination	Nucleic acid detection for point-of-care molecular diagnostics	[18]

**Table 3:** Comparison of Cas9, Cas12, Cas13, and Cas14 proteins.

Cas protein effector	Conducting the way	Length of interval sequence	PAM/PFS	Recognition of substrates	Cutting the substrate	Enzyme structural domain
Cas9	sgRNA	18–24 nt	3′, NGG	dsDNA	dsDNA	HNH, RuvC
Cas12	crRNA	18–24 nt	3′, TTTN	dsDNA, ssDNA	ssDNA	RuvC-like
Cas13	crRNA	22–28 nt	3′, Non-G-PFS	ssRNA	ssRNA	2X HEPN
Cas14	sgRNA	2–40 nt	None	ssRNA	ssDNA	RuvC-like

*Note:* sgRNA: single-guide RNA; sgRNA is naturally occurring RNA derived from the bacterial CRISPR system that directs Cas proteins to cut specific DNA by providing recognition of the target DNA sequence. crRNA: CRISPR RNA; crRNA is synthetic RNA that combines the functions of crRNA and tracrRNA (trans-activating CRISPR RNA), simplifying the use of the CRISPR-Cas system and making gene editing more efficient and controllable.

**Table 4:** CRISPR-Cas system–based detection of SARS-CoV-2 variants.

Effect protein	Method of detection	Method of amplification	Location of mutation	Sensitivity (95% CI)	Assay reaction time	LoD (95% CI)	Mode of read out	References
Cas 9	FELUDA	RT-RPA	L452R, T478K, N501Y	100%	1 h	4.0 × 10^5^ copies/mL	Fluorescence signal	[38–40]
	Bio-SCAN	RT-RPA	N501Y, E484K		1 h		Naked eye color matching	[38, 39, 41]

Cas 12	On-site detection method for the SARS-CoV-2 E484K	RT-PCR/RT-RPA	E484K	95%	Less than 70 min	1.0 × 10^4^ copies/mL	Fluorescence signal	[33, 39, 42]
	RT-CORDS	RT-PCR	N501Y, D614G		30–60 min		Naked eye color matching	[31, 39, 42]

Cas 13	miSHERLOCK	RT-RPA	N501Y, E484K, L452R	100%	1 h	4.2 × 10^4^ copies/mL	Fluorescence signal	[39, 43, 44]
	APHF-analyzer	RT-RPA	N501Y, L452R		30 min		Naked eye color matching	[32, 39, 43]

Cas 14	Cas14-DETECTR	RPA	N501Y, E484K	95%	1–2 h	0.4 × 10^4^ copies/mL	Fluorescence signal	[39, 45]

*Note:* These LoD values were all determined in a system with a reaction volume of 0.02 mL.

Abbreviations: 95% CI, 95% confidence interval; LoD, limit of detection.

**Table 5:** The time frame required and accuracy rates for various diagnostic approaches.

Detection method	Result time	LoD (copies/mL)	Sensitivity (%)	Specificity (%)	References
mNGS	4–6 h	100,000	88	100	[72]
RT-fqPCR	45–120 min	100–75,000	100	100	[16]
ddPCR	2–4 h	625	94	100	[17]
DETECTR	40 min	10,000	90	100	[73]
SHERLOCK	1 h	900	95	100	[73]
FIND-IT	30 min	100,000	95	100	[18, 62]

## Data Availability

Data sharing is not applicable to this article as no datasets were generated or analyzed during the current study.
